# Supplementary material for: Inhibition of casein kinase 2 induces cell death in tyrosine kinase inhibitor resistant chronic myelogenous leukemia cells
Source: PLoS One. 2023 May 4;18(5):e0284876. doi: 10.1371/journal.pone.0284876 (PMC10159124; doi:10.1371/journal.pone.0284876)
Supplement: S1 File — (PDF) [file pone.0284876.s001.pdf]

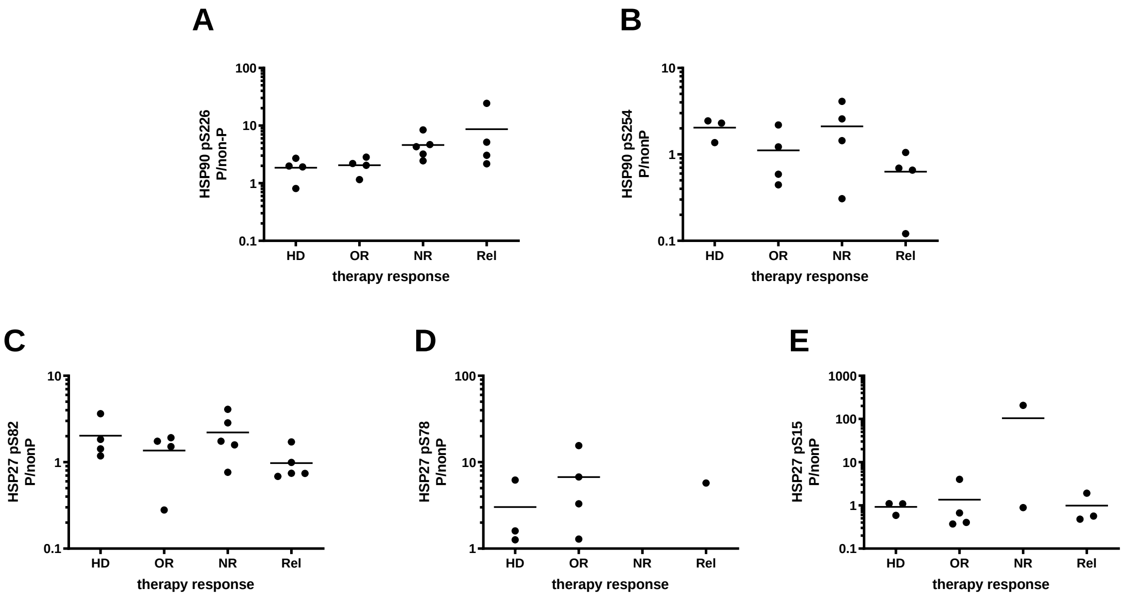

Supplementary figure S1. HSP90 serine 226 phosphorylation levels in CML patients with differing responses to therapy.

Protein-antibody array analysis of 14 samples from patients with a different response to therapy. The ratios of phosphorylated and non-phosphorylated indicated serine residues are given. Therapy response: HD — healthy donor, OR — optimal response, NR — nonresponding, Rel — relapsed.

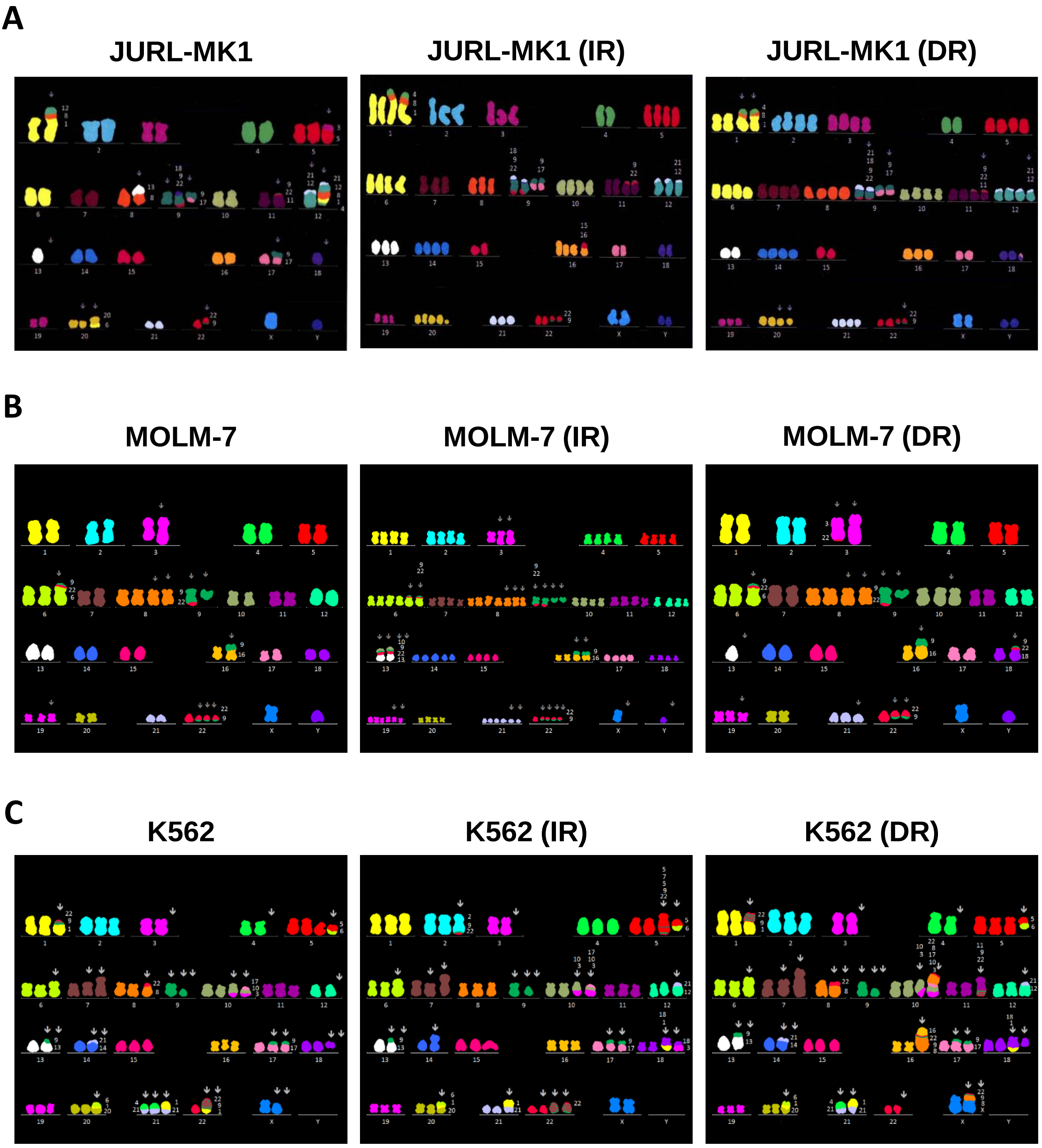

Supplementary figure S2. Cytogenetic analysis of TKI-resistant cells. Multicolor fluorescence in situ hybridization (mFISH) were used to characterize the karyotype of all CML-derived cells and their IR and DR sublines. (A) JURL-MK1, (B) MOLM-7, (C) K562. IR—imatinib resistant, DR—dasatinib resistant.

Supplementary figure S2. Cytogenetic analysis of TKI-resistant cells (continuation).

In all JURL-MK1 derived cells, chromosomal aberrations were almost identical to their parental line. Only polyploid mitoses were present, and the BCR-ABL fusion signal was detected on the same chromosomes in all the three JURL-MK1 cell lines (Supplementary Figure S3A).

In TKI-sensitive MOLM-7 cells (Supplementary Figure S3B), we detected structural changes relating to the BCR-ABL fusion gene. The control cells had two extra numerical Ph chromosomes. MOLM-7 IR cells were shown to be polyploid, and all BCR-ABL signals were present in two copies. One extra numerical Ph was present, and a second one was translocated and amplified on the short arm of a derivative chromosome 13. In MOLM-7 DR cells, again, one extra numerical Ph chromosome was detected, and a second one was translocated to the short arm of chromosome 18. Additionally, in all MOLM-7 cells, the BCR-ABL fusion signal was also detected on the short arm of chromosome 6.

The K562 cells used in this study are near triploid and posess characteristic markers as previously reported (27,28). One copy of the chromosomes X, 3, 9, 13, and 14 was lost in all K562 cells. In all the three K562 sub-lines the same abnormalities were present on several chromosomes (Supplementary Figure S3C). Additionally, some differences were detected. Results of fluorescence in-situ hybridization (FISH) with BCR-ABL specific probes showed that K562 and K562 DR (but not K562 IR) had BCR-ABL fusion on the short arm of the derivative chromosome 1. In K562 and K562 IR (but not K562 DR), this aberration was also found on the long arm of the derivative chromosome 22. In K562 IR, other non-amplified BCR-ABL fusions were detected on the long arms of the derivative chromosomes 2 and 5. In K562 DR, an additional BCR-ABL amplified signal was detected on the long arm of the derivative chromosome 11, and a non-amplified signal in the long arm of chromosome 16 and on the short arm of the chromosome X.

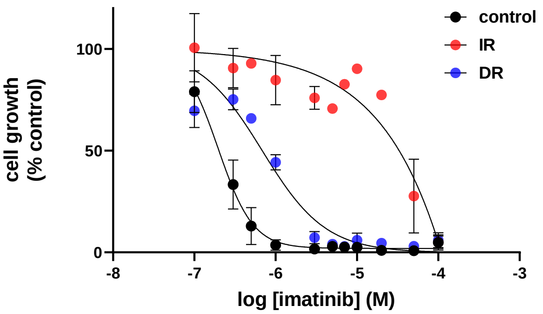

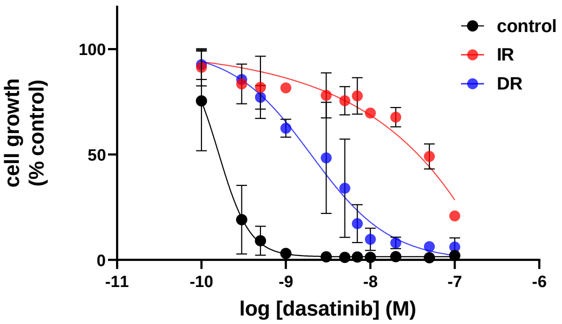

Supplementary figure S3. EC50 values of imatinib and dasatinib effect on sensitive and resistant cells proliferation.

(A) EC50 values of imatinib and dasatinib on all tested cells. Cell lines were incubated with imatinib (range 0 - 100 µM) or dasatinib (range 0 - 100 nM). EC50 was defined as the TKI concentration that caused 50% reduction in proliferation/viability (as assessed by Alamar blue method). EC50 values and confidence intervals (CI95%) were calculated from 2-3 independent experiments. Representative dose-response curves are presented for imatinib (B) and dasatinib (C) tested in JURL-MK1 cells (ctrl) and their resistant sub-lines. Graphs are means from 2-3 independent experiments, bars = s.d. IR — imatinib-resistant cells, DR — dasatinib-resistant cells.

Supplementary table 1A. Mutational status of BCR-ABL kinase domain in JURL-MK1 cells.
The results of Sanger sequencing of the BCR-ABL kinase domain are included in both forward (F) and reverse (R) sense

Cell line	BCR-ABL Kinase domain sequence (Forward + Reverse)
JURL-MK1	F:<br>CGACAAGTGGGAGATGGAACGCACGGACATCACCATGAAGCACAAGCTGGGCGGGGGCCAGTACGGGGAGGTGTAC<br>GAGGGCGTGTGGAAGAAATACAGCCTGACGGTGGCCGTGAAGACCTTGAAGGAGGACACCATGGAGGTGGAAGAGT<br>TCTTGAAAGAAGCTGCAGTCATGAAAGAGATCAAACACCCTAACCTGGTGCAGCTCCTTGGGGTCTGCACCCGGGA<br>GCCCCCGTTCTATATCATCACTGAGTTCATGACCTACGGGAACCTCCTGGACTACCTGAGGGAGTGCAACCGGCAG<br>GAGGTGAACGCCGTGGTGCTGCTGTACATGGCCACTCAGATCTCGTCAGCCATGGAGTACCTGGAGAAGAAAAACT<br>TCATCCACAGAGATCTTGCTGCCCGAAACTGCCTGGTAGGGGAGAACCACTTG<br>R:<br>GTGAAGGTAGCTGATTTTGGCCTGAGCAGGTTGATGACAGGGGACACCTACACAGCCCATGCTGGAGCCAAGTTCC<br>CCATCAAATGGACTGCACCCGAGAGCCTGGCCTACAACAAGTTCTCCATCAAGTCCGACGTCTGGGCATTTGGAGT<br>ATTGCTTTGGGAAATTGCTACCTATGGCATGTCCCCTTACCCGGGAATTGACCTGTCCCAGGTGTATGAGCTGCTA<br>GAGAAGGACTACCGCATGGAGCGCCCAGAAGGCTGCCCAGAGAAGGTCTATGAACTCATGCGAGCATGTTGGCAGT<br>GGAATCCCTCTGACCGGCCCTCCTTTGCTGAAATCCACCAAGCCTTtgaA
JURL-MK1 IR	F:<br>CGACaAGTGGGAGATGGAaCGCACGGACATCACCATGAAGCACAAGCTGGGCGGGGGCCAGTACGGgGAGGTGTAC<br>GAGGGCGTGTGGAAGAAATACAGCCTGACGGTGGCCGTGAAGACCTTGAAGGAGGACACCATGGAGGTGGAAGAGT<br>TCTTGAAAGAAGCTGCAGTCATGAAAGAGATCAAACACCCTAACCTGGTGCAGCTCCTTGGGGTCTGCACCCGGGA<br>GCCCCCGTTCTATATCATCAYTGAGTTCATGACCTACGGGAACCTCCTGGACTACCTGAGGGAGTGCAACCGGCAG<br>GAGGTGAACGCCGTGGTGCTGCTGTACATGGCCACTCAGATCTCGTCAGCCATGGAGTACCTGGAGAAGAAAAACT<br>TCATCCACAGAGATCTTGCTGCCCGAAACTGCCTGGTAGGGGAGAACCACTTG<br>R:<br>GTGAAGGTAGCTGATTTTGGCCTGAGCAGGTTGATGACAGGGGACACCTACACAGCCCATGCTGGAGCCAAGTTCC<br>CCATCAAATGGACTGCACCCGAGAGCCTGGCCTACAACAAGTTCTCCATCAAGTCCGACGTCTGGGCATTTGGAGT<br>ATTGCTTTGGGAAATTGCTACCTATGGCATGTCCCCTTACCCGGGAATTGACCTGTCCCAGGTGTATGAGCTGCTA<br>GAGAAGGACTACCGCATGGAGCGCCCAGAAGGCTGCCCAGAGAAGGTCTATGAACTCATGCGaGCATGTTGGCAGT<br>GGAATCCCTCTGACCGGCCCTCCTTTGCTGAAATCCACCAAGCCTTTgA
JURL-MK1 DR	F:<br>CGACAAGTGGGAGATGGAACGCACGGACATCACCATGAAGCACAAGCTGGGCGGGGGCCAGTACGGGGAGGTGTAC<br>GAGGGCGTGTGGAAGAAATACAGCCTGACGGTGGCCGTGAAGACCTTGAAGGAGGACACCATGGAGGTGGAAGAGT<br>TCTTGAAAGAAGCTGCAGTCATGAAAGAGATCAAACACCCTAACCTGGTGCAGCTCCTTGGGGTCTGCACCCGGGA<br>GCCCCCGTTCTATATCATCAYTGAGTTCATGACCTACGGGAACCTCCTGGACTACCTGAGGGAGTGCAACCGGCAG<br>GAGGTGAACGCCGTGGTGCTGCTGTACATGGCCACTCAGATCTCGTCAGCCATGGAGTACCTGGAGAAGAAAAACT<br>TCATCCACAGAGATCTTGCTGCCCGAAACTGCCTGGTAGGGGAGAACCACTTG<br>R:<br>GTGAAGGTAGCTGATTTTGGCCTGAGCAGGTTGATGACAGGGGACACCTACACAGCCCATGCTGGAGCCAAGTTCC<br>CCATCAAATGGACTGCACCCGAGAGCCTGGCCTACAACAAGTTCTCCATCAAGTCCGACGTCTGGGCATTTGGAGT<br>ATTGCTTTGGGAAATTGCTACCTATGGCATGTCCCCTTACCCGGGAATTGACCTGTCCCAGGTGTATGAGCTGCTA<br>GAGAAGGACTACCgCaTGgaGCGCCCAGAAGGCTGCCCAGAGAAGGTCTATGAACTCATGCGAGCATGTTGGCAGT<br>GGAATCCCTCTGACCGGCCCTCCTTTGCTGAAATCCACCAAGCCTTttgAA

Supplementary table 1B. Mutational status of BCR-ABL kinase domain in MOLM-7 cells.
The results of Sanger sequencing of the BCR-ABL kinase domain are included in both forward (F) and reverse (R) sense.

Cell line	BCR-ABL Kinase domain sequence (Forward + Reverse)
MOLM-7	F:<br><br>GACAAGTGGGAGATGGAACGCACGGACATCACCATGAAGCACAAGCTGGGCGGGGGCCAGTACGGggAGGTGTACG<br>AGGGCGTGTGGAAGAAATACAGCCTGACGGTGGCCGTGAAGACCTTGAAGGAGGACACCATGGAGGTGGAAGAGTT<br>CTTGAAAGAAGCTGCAGTCATGAAAGAGATCAAACACCCTAACCTGGTGCAGCTCCTTGGGGTCTGCACCCGGGAG<br>CCCCCGTTCTATATCATCACTGAGTTCATGACCTACGGGAACCTCCTGGACTACCTGAGGGAGTGCAACCGGCAGG<br>AGGTGAACGCCGTGGTGCTGCTGTACATGGCCACTCAGATCTCGTCAGCCATGGAGTACCTGGAGAAGAAAAACTT<br>CATCCACAGAGATCTTGCTGCCCGAAACTGCC<br><br>R:<br><br>TGGTAGGGGAGAACCACTTGGTGAAGGTAGCTGATTTTGGCCTGAGCAGGTTGATGACAGGGGACACCTACACAGC<br>CCATGCTGGAGCCAAGTTCCCCATCAAATGGACTGCACCCGAGAGCCTGGCCTACAACAAGTTCTCCATCAAGTCC<br>GACGTCTGGGCATTTGGAGTATTGCTTTGGGAAATTGCTACCTATGGCATGTCCCCTTACCCGGGAATTGACCTGT<br>CCCAGGTGTATGAGCTGCTAGAGAAGGACTACCGCATGGAGCGCCCAGAAGGCTGCCCAGAGAAGGTCTATGAACT<br>CATGCgAGCATGTTGGCAGTGGAATCCCTCTGACCGGCCCTCCTTTGCTGAAATCCACCAAGCCTTTGAA
MOLM-7 IR	F:<br><br>CGACAAGTGGGAGATGGAACGCACGGACATCACCATGAAGCACAAGCTGGGCGGGGGCCAGTACGGGGAGGTGTAC<br>GAGGGCGTGTGGAAGAAATACAGCCTGACGGTGGCCGTGAAGACCTTGAAGGAGGACACCATGGAGGTGGAAGAGT<br>TCTTGAAAGAAGCTGCAGTCATGAAAGAGATCAAACACCCTAACCTGGTGCAGCTCCTTGGGGTCTGCACCCGGGA<br>GCCCCCGTTCTATATCATCACTGAGTTCATGACCTACGGGAACCTCCTGGACTACCTGAGGGAGTGCAACCGGCAG<br>GAGGTGAACGCCGTGGTGCTGCTGTACATGGCCACTCAGATCTCGTCAGCCATGGAGTACCTGGAGAAGAAAAACT<br>TCATCCACAGAGATCTTGCTGCCCGAAACTGCCTGGTAGGGGAGAACCACTTG<br><br>R:<br><br>GTGAAGGTAGCTGATTTTGGCCTGAGCAGGTTGATGACAGGGGACACCTACACAGCCCATGCTGGAGCCAAGTTCC<br>CCATCAAATGGACTGCACCCGAGAGCCTGGCCTACAACAAGTTCTCCATCAAGTCCGACGTCTGGGCATTTGGAGT<br>ATTGCTTTGGGAAATTGCTACCTATGGCATGTCCCCTTACCCGGGAATTGACCTGTCCCAGGTGTATGAGCTGCTA<br>GAGAAGGACTACCGCATGGAGCGCCCAGAAGGCTGCCCAGAGAAGGTCTATGAACTCATGCGAGCATGTTGGCAGT<br>GGAATCCCTCTGACCGGCCCTCCTTTGCTGAAATCCACCAAGCCTTTGA
MOLM-7 DR	F:<br><br>CGACAAGTGGGAGATGGAACGCACGGACATCACCATGAAGCACAAGCTGGGCGGGGGCCAGTACGGGGAGGTGTAC<br>GAGGGCGTGTGGAAGAAATACAGCCTGACGGTGGCCGTGAAGACCTTGAAGGAGGACACCATGGAGGTGGAAGAGT<br>TCTTGAAAGAAGCTGCAGTCATGAAAGAGATCAAACACCCTAACCTGGTGCAGCTCCTTGGGGTCTGCACCCGGGA<br>GCCCCCGTTCTATATCATCACTGAGTTCATGACCTACGGGAACCTCCTGGACTACCTGAGGGAGTGCAACCGGCAG<br>GAGGTGAACGCCGTGGTGCTGCTGTACATGGCCACTCAGATCTCGTCAGCCATGGAGTACCTGGAGAAGAAAAACT<br>TCATCCACAGAGATCTTGCTGCCCGAAACTGCCTGGTAGGGGAGAACCACTTG<br><br>R:<br><br>GTGAAGGTAGCTGATTTTGGCCTGAGCAGGTTGATGACAGGGGACACCTACACAGCCCATGCTGGAGCCAAGTTCC<br>CCATCAAATGGACTGCACCCGAGAGCCTGGCCTACAACAAGTTCTCCATCAAGTCCGACGTCTGGGCATTTGGAGT<br>ATTGCTTTGGGAAATTGCTACCTATGGCATGTCCCCTTACCCGGGAATTGACCTGTCCCAGGTGTATGAGCTGCTA<br>GAGAAGGACTACCGCATGGAGCGCCCAGAAGGCTGCCCAGAGAAGGTCTATGAACTCATGCGAGCATGTTGGCAGT<br>GGAATCCCTCTGACCGGCCCTCCTTTGCTGAAATCCACCAAGCCtTtgA

Supplementary table 1C. Mutational status of BCR-ABL kinase domain in K562 cells.
The results of Sanger sequencing of the BCR-ABL kinase domain are included in both forward (F) and reverse (R) sense.

Cell line	BCR-ABL Kinase domain sequence (Forward + Reverse)
K562	F:<br><br>CGACAAGTGGGAGATGGAACGCACGGACATCACCATGAAGCACAAGCTGGGCGGGGGCCAGTACGGGGAGGTGTAC<br>GAGGGCGTGTGGAAGAAATACAGCCTGACGGTGGCCGTGAAGACCTTGAAGGAGGACACCATGGAGGTGGAAGAGT<br>TCTTGAAAGAAGCTGCAGTCATGAAAGAGATCAAACACCCTAACCTGGTGCAGCTCCTTGGGGTCTGCACCCGGGA<br>GCCCCCGTTCTATATCATCACTGAGTTCATGACCTACGGGAACCTCCTGGACTACCTGAGGGAGTGCAACCGGCAG<br>GAGGTGAACGCCGTGGTGCTGCTGTACATGGCCACTCAGATCTCGTCAGCCATGGAGTACCTGGAGAAGAAAAACT<br>TCATCCACAGAGATCTTGCTGCCCGAAACTGCCTGGTAGGGGAGAACCACTTG<br><br>R:<br><br>GTGAAGGTAGCTGATTTTGGCCTGAGCAGGTTGATGACAGGGGACACCTACACAGCCCATGCTGGAGCCAAGTTCC<br>CCATCAAATGGACTGCACCCGAGAGCCTGGCCTACAACAAGTTCTCCATCAAGTCCGACGTCTGGGCATTTGGAGT<br>ATTGCTTTGGGAAATTGCTACCTATGGCATGTCCCCTTACCCGGGAATTGACCTGTCCCAGGTGTATGAGCTGCTA<br>GAGAAGGACTACCGCATGGAGCGCCCAGAAGGCTGCCCAGAGAAGGTCTATGAACTCATGCGAGCATGTTGGCAGT<br>GGAATCCCTCTGACCGGCCCTCCTTTGCTGAAATCCACCAAGCCTTTGAA
K562 IR	F:<br><br>CGACAAGTGGGAGATGGAACGCACGGACATCACCATGAAGCACAAGCTGGGCGGGGGCCAGTACGGGGAGGTGTAC<br>GAGGGCGTGTGGAAGAAATACAGCCTGACGGTGGCCGTGAAGACCTTGAAGGAGGACACCATGGAGGTGGAAGAGT<br>TCTTGAAAGAAGCTGCAGTCATGAAAGAGATCAAACACCCTAACCTGGTGCAGCTCCTTGGGGTCTGCACCCGGGA<br>GCCCCCGTTCTATATCATCACTGAGTTCATGACCTACGGGAACCTCCTGGACTACCTGAGGGAGTGCAACCGGCAG<br>GAGGTGAACGCCGTGGTGCTGCTGTACATGGCCACTCAGATCTCGTCAGCCATGGAGTACCTGGAGAAGAAAAACT<br>TCATCCACAGAGATCTTGCTGCCCGAAACTGCCTGGTAGGGGAGAACCACTTG<br><br>R:<br><br>GTGAAGGTAGCTGATTTTGGCCTGAGCAGGTTGATGACAGGGGACACCTACACAGCCCATGCTGGAGCCAAGTTCC<br>CCATCAAATGGACTGCACCCGAGAGCCTGGCCTACAACAAGTTCTCCATCAAGTCCGACGTCTGGGCATTTGGAGT<br>ATTGCTTTGGGAAATTGCTACCTATGGCATGTCCCCTTACCCGGGAATTGACCTGTCCCAGGTGTATGAGCTGCTA<br>GAGAAGGACTACCGCATGGAGCGCCCAGAAGGCTGCCCAGAGAAGGTCTATGAACTCATGcgAGCATGTTGGCAGT<br>GGAATCCCTCTGACCGGCCCTCCTTTGCTGAAATCCACCAAGCCTTTGAA
K562 DR	F:<br><br>CGACAaGTGGGAGATGGAACGCACGGACATCACCATGAAGCACAAGCTGGGCGGGGGCCAGTACGGGGAGGTGTAC<br>GAGGGCGTGTGGAAGAAATACAGCCTGACGGTGGCCGTGAAGACCTTGAAGGAGGACACCATGGAGGTGGAAGAGT<br>TCTTGAAAGAAGCTGCAGTCATGAAAGAGATCAAACACCCTAACCTGGTGCAGCTCCTTGGGGTCTGCACCCGGGA<br>GCCCCCGTTCTATATCATCACTGAGTTCATGACCTACGGGAACCTCCTGGACTACCTGAGGGAGTGCAACCGGCAG<br>GAGGTGAACGCCGTGGTGCTGCTGTACATGGCCACTCAGATCTCGTCAGCCATGGAGTACCTGGAGAAGAAAAACT<br>TCATCCACAGAGATCTTGCTGCCCGAAACTGCCTGGTAGGGGAGAACCACTTG<br><br>R:<br><br>GTGAAGGTAGCTGATTTTGGCCTGAGCAGGTTGATGACAGGGGACACCTACACAGCCCATGCTGGAGCCAAGTTCC<br>CCATCAAATGGACTGCACCCGAGAGCCTGGCCTACAACAAGTTCTCCATCAAGTCCGACGTCTGGGCATTTGGAGT<br>ATTGCTTTGGGAAATTGCTACCTATGGCATGTCCCCTTACCCGGGAATTGACCTGTCCCAGGTGTATGAGCTGCTA<br>GAGAAGGACTACCGCATGGAGCGCCCAGAAGGCTGCCCAGAGAAGGTCTATGAACTCATGCGAGCATGTTGGCAGT<br>GGAATCCCTCTGACCGGCCCTCCTTTGCTGAAATCCACCAAGCCTTTGAA

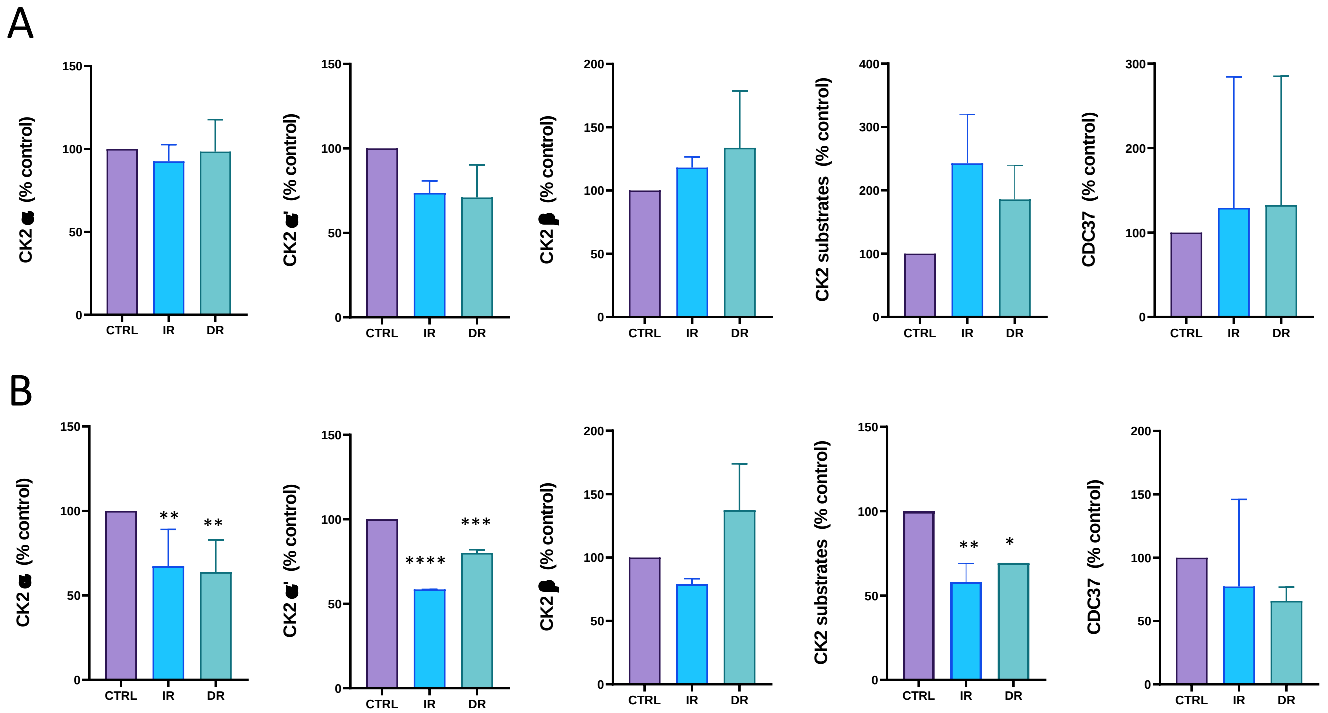

Supplementary figure S4. Protein levels of CK2 subunits and its substrates phosphorylation in cell lines MOLM-7 (A) and K562 (B) and resistant their sub-lines.
Densitometric graphs were calculated from western blots with appropriate antibodies. Phosphorylation and protein levels were normalised to β-actin and related to control. Means and standard deviation obtained from at least 3 biological replicates are shown. (***P < 0.001, **P<0.01, *P<0.1).

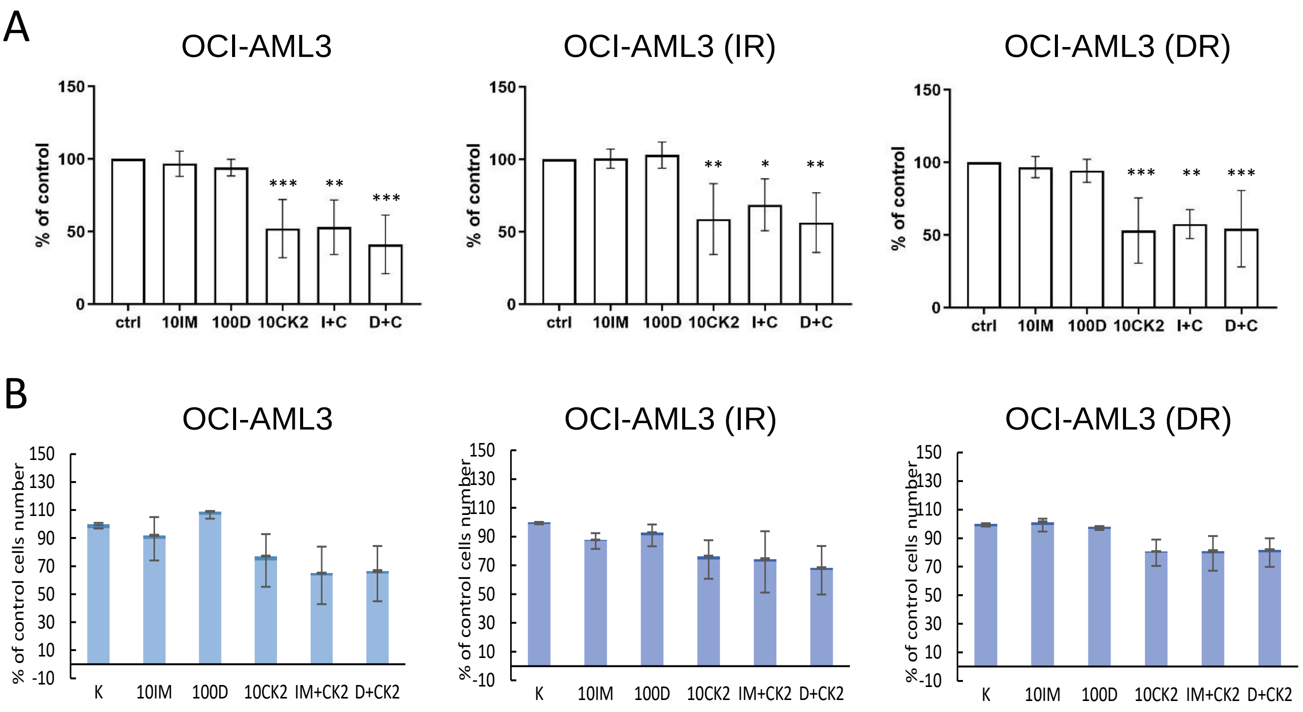

Supplementary Figure S5. Effects of CX-4945 on cell proliferation and viability BCR-ABL negative OCI-AML3 cells.

The cells were treated for 48 h with imatinib (IM, 10µM), dasatinib (D, 100nM), CX-4945 (CK, 10µM), and their combinations. (A) Proliferation/cell activity of OCI-AML3 cells was assessed by the Alamar blue method and related to the control. The data represents mean with SD of 3 – 6 separate experiments, statistical significance was assessed by one-way ANOVA followed by a Dunnett's multiple comparisons test (B) Cell numbers and viability were evaluated by trypan blue staining. Light bars—live cells, dark bars—dead cell fraction.

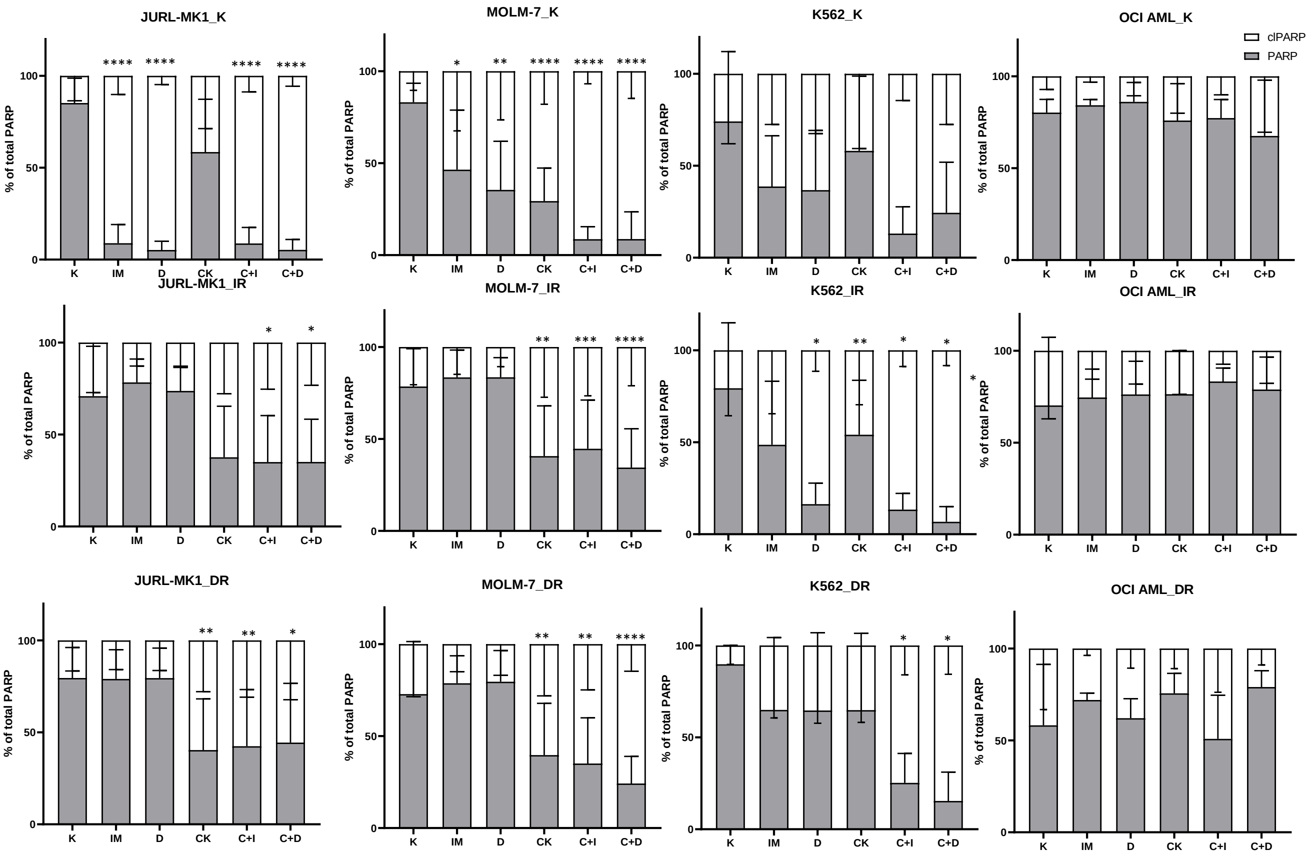

Supplementary Figure S6. Effects of CX-4945 on the PARP cleavage.

Densitometric evaluation of PARP/clPARP. Means and standard deviation obtained from at least 5 experiments (3 biological replicates, each at least 2 times analysed in western blot) are shown. ****P<0.0001, ***P < 0.001, **P<0.01, *P<0.05. C – control, IR – imatinib-resistant, DR – dasatinib-resistant.
