# Supplementary material for: Inhibition of casein kinase 2 induces cell death in tyrosine kinase inhibitor resistant chronic myelogenous leukemia cells
Source: PLoS One. 2023 May 4;18(5):e0284876. doi: 10.1371/journal.pone.0284876 (PMC10159124; doi:10.1371/journal.pone.0284876)
Supplement: S1 Raw images — (PDF) [file pone.0284876.s002.pdf]

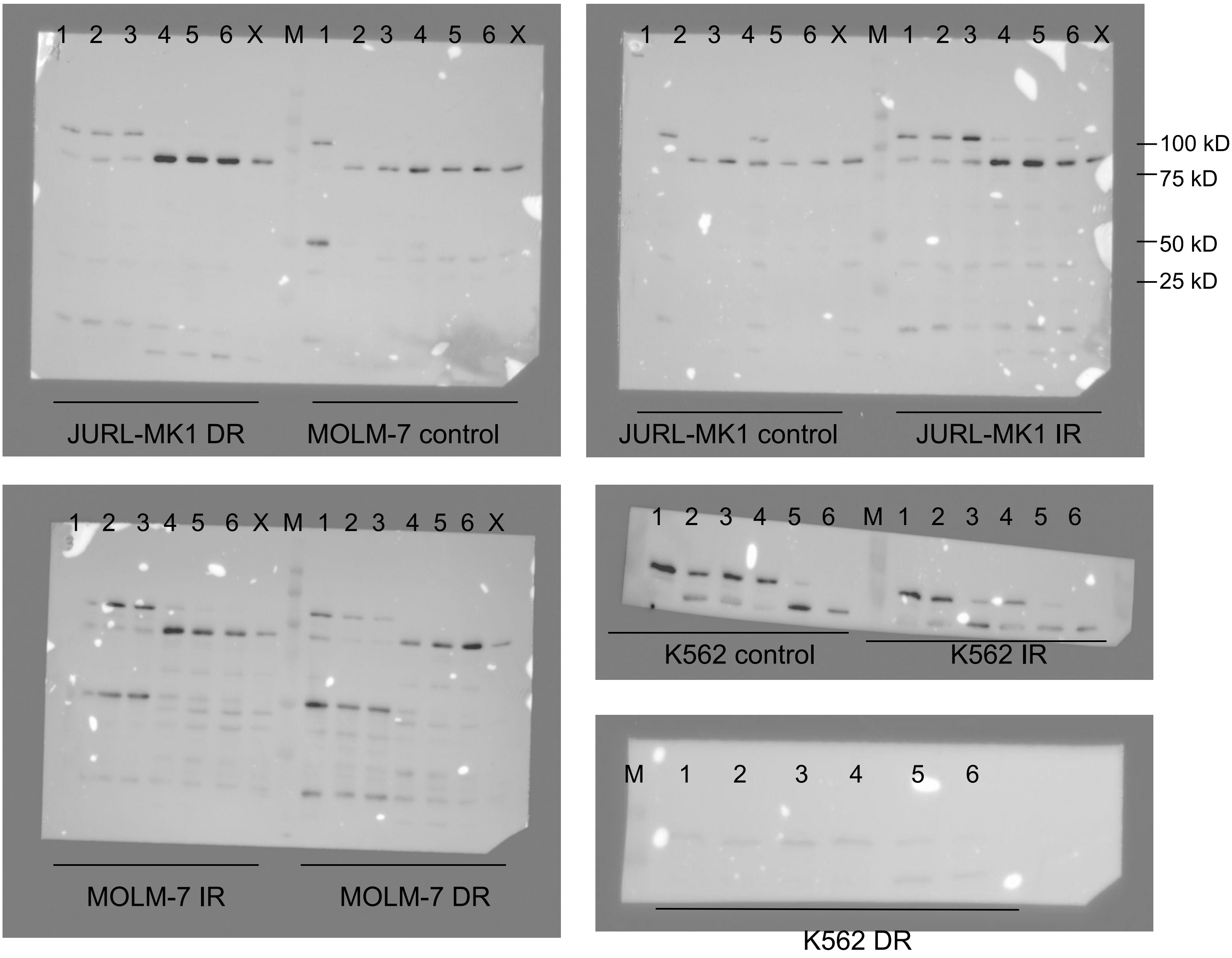

Original WB images used to compile Fig3C.
The cell lines used are indicated below each sample group. The samples were as follows: (1) control (2) imatinib, 10 μM (3) dasatinib, 100 nM (4) CX-4945, 10 μM (5) imatinib + CX-4945 (6) dasatinib + CX-4945 (X) sample excluded from the figure (M) Mw marker. Molecular weights of the major bands in Mw marker are indicated. The images were acquired as described in the methods section of the manuscript.
Please notice that some membranes were cut due to antibody preservation (K562 control, IR, DR), and we provide images of the whole cut.
